# The role of fibrosis, inflammation, and congestion biomarkers for outcome prediction in candidates to cardiac resynchronization therapy: is “response” the right answer?

**DOI:** 10.3389/fcvm.2023.1180960

**Published:** 2023-06-12

**Authors:** Matteo Beltrami, Alessandro Galluzzo, Riccardo Tappa Brocci, Alessandro Paoletti Perini, Paolo Pieragnoli, Manuel Garofalo, Geza Halasz, Massimo Milli, Maria Barilli, Alberto Palazzuoli

**Affiliations:** ^1^ Cardiology Unit, San Giovanni di Dio Hospital, Azienda USL Toscana Centro, Florence, Italy; ^2^ Cardiology Unit, Santa Croce Hospital, Moncalieri, Italy; ^3^ Department of Clinical Trial, Le Scotte Hospital, University of Siena, Siena, Italy; ^4^ Department of Internal Medicine, Cardiology and Electrophysiology Unit, Azienda USL Toscana Centro, Florence, Italy; ^5^ Arrhythmia and Electrophysiology Unit, Careggi University Hospital, Florence, Italy; ^6^ Department of Clinical and Experimental Medicine, Careggi University Hospital, Florence, Italy; ^7^ Department of Cardiosciences, Azienda Ospedaliera San Camillo-Forlanini, Rome, Italy; ^8^ Department of Medical Biotechnologies, Division of Cardiology, University of Siena, Le Scotte Hospital, Siena, Italy; ^9^ Cardiovascular Diseases Unit, Cardio Thoracic and Vascular Department, Le Scotte Hospital, University of Siena, Siena, Italy

**Keywords:** galectin-3, sST2, eGFR, biomarkers, heart failure, outcome, HF hospitalization, cardiovascular death

## Abstract

**Background:**

Cardiac resynchronization therapy (CRT) is an established treatment in selected patients suffering from heart failure with reduced ejection fraction (HFrEF). It has been proposed that myocardial fibrosis and inflammation could influence CRT “response” and outcome. Our study investigated the long-term prognostic significance of cardiac biomarkers in HFrEF patients with an indication for CRT.

**Methods:**

Consecutive patients referred for CRT implantation were retrospectively evaluated. The soluble suppression of tumorigenicity 2 (sST2), galectin-3 (Gal-3), N-terminal portion of the B-type natriuretic peptide (NT-proBNP), and estimated glomerular filtration rate (eGFR) were measured at baseline and after 1 year of follow-up. Multivariate analyses were performed to evaluate their correlation with the primary composite outcome of cardiovascular mortality and heart failure hospitalizations at a mean follow-up of 9 ± 2 years.

**Results:**

Among the 86 patients enrolled, 44% experienced the primary outcome. In this group, the mean baseline values of NT-proBNP, Gal-3, and sST2 were significantly higher compared with the patients without cardiovascular events. At the multivariate analyses, baseline Gal-3 [cut-off: 16.6 ng/ml, AUC: 0.91, *p* < 0.001, HR 8.33 (1.88–33.33), *p* = 0.005] and sST2 [cut-off: 35.6 ng/ml AUC: 0.91, *p* < 0.001, HR 333 (250–1,000), *p* = 0.003] significantly correlated with the composite outcome in the prediction models with high likelihood. Among the parameters evaluated at 1-year follow-up, sST2, eGFR, and the variation from baseline to 1-year of Gal-3 levels showed a strong association with the primary outcome [HR 1.15 (1.08–1.22), *p* < 0.001; HR: 0.84 (0.74–0.91), *p* = 0.04; HR: 1.26 (1.10–1.43), *p* ≤ 0.001, respectively]. Conversely, the echocardiographic definition of CRT response did not correlate with any outcome.

**Conclusion:**

In HFrEF patients with CRT, sST2, Gal-3, and renal function were associated with the combined endpoint of cardiovascular death and HF hospitalizations at long-term follow-up, while the echocardiographic CRT response did not seem to influence the outcome of the patients.

## 1. Introduction

Despite the significant advances in medical treatment, the prognosis in heart failure with reduced ejection fraction (HFrEF) remains poor, and the use of markers for outcome prediction remains scarce. Cardiac resynchronization therapy (CRT) proved to reduce mortality and heart failure (HF) hospitalizations in patients with left bundle branch block (LBBB) and left ventricular ejection fraction (LVEF) ≤35%, still symptomatic on top of optimal medial therapy (1). However, given that not all patients seem to equally benefit from CRT, the concept of “response” has been developed: various definitions, mainly based on clinical or echocardiographic modifications following CRT implantation, have tried to identify the subgroup of HF patients that gains the greatest advantage from resynchronization therapy. The aim is to optimize the candidates’ selection and the cost/benefit ratio of a relatively expensive tool (2). The degree of myocardial inflammation and fibrosis can impair the efficiency of resynchronization by affecting left ventricle (LV) adverse remodeling and outcome, becoming the main determinant of the so-called “CRT response” (3). Clinical and imaging assessments alone, performed before CRT implantation, are not able to fully evaluate the state of cardiomyocytes and myocardial extracellular matrix. Conversely, some biomarkers, such as the soluble suppression of tumorigenicity 2 (sST2), galectin-3 (Gal-3), and N-terminal portion of the B-type natriuretic peptide (NT-proBNP), have been related with myocardial fibrosis, inflammation, and congestion, which are affecting the prognosis in HF patients (4–6). Chronic kidney disease and HF may amplify pathophysiologic mechanisms that lead to a dangerous vicious cycle. It is still unclear whether the dynamic change of renal function after CRT implantation directly contributes to a poor outcome or whether eGFR only marks the advances of cardiac and renal dysfunction (7).

The associations between the variations of the mentioned biomarkers, renal function, CRT response, and cardiovascular (CV) outcome have not been systematically evaluated in contemporary cohorts. Thus, the aim of our study is to investigate the potential relationship of cardiac biomarkers, CRT response, and long-term outcome in a cohort of patients with HFrEF undergoing CRT implantation.

## 2. Materials and methods

### 2.1. Study design and participants

We retrospectively evaluated consecutive patients undergoing implantation of CRT pacing (CRT-P) or CRT and defibrillation (CRT-D) in our institution “Azienda Ospedaliera-Universitaria Careggi” from November 2010 to January 2012. According to current guidelines, the patients were addressed for implantation when affected by symptomatic HFrEF (New York Heart Association class II to ambulatory class IV) despite optimal medical therapy, LV systolic dysfunction with ejection fraction ≤35%, and QRS width ≥130 ms together with LBBB morphology (8). The presence of a LBBB was defined in case of QRS ≥ 130 ms; QS or rS complex in V1 to V2; monophasic and notched or slurred R waves in I, aVL, V5, or V6; and absent Q waves in leads V5 and V6 (9). A three pacing-lead device was implanted in each patient and was programmed to obtain the highest percentage of biventricular stimulation (≥90% of total beats). This study excluded patients with a QRS morphology different from LBBB or already carriers of a right-sided pacing system, either pacemaker or implantable defibrillator. The implantation of transvenous CRT systems was performed according to standard techniques, preferring the basal position of the lateral veins for LV lead placement avoiding the apical segment (10), and placing the right atrial and ventricular leads preferably at the atrial appendage and at the apex (11). No quadripolar LV leads were implanted since they were not available at that time in our institution. The CRTs were programmed by senior electrophysiology specialists according to current guidelines and manufacturer specifications (12). Our study is in accordance with the ethical guidelines of the 1975 Declaration of Helsinki and was approved by our local institutional review board. Informed consents were obtained from all the patients.

### 2.2. Laboratory assessment

Gal-3, sST2, NT-proBNP, creatinine, and estimated glomerular filtration rate (eGFR calculated with the CKD-EPI formula) were measured at baseline and at 12 months after CRT implantation. The delta (*Δ*) was considered as the difference between the biomarkers at baseline and 1-year follow-up.

All blood samples obtained from the patients were collected with a sterile disposable syringe containing EDTA. They were analyzed using the Alere Triage BNP Test. This test is an immunoassay in a single-use plastic cartridge containing a monoclonal antibody for BNP, labeled with a fluorescent dye and BNP. Plasma BNP was measured with Triage BNP Test (Biosite Inc., San Diego, CA, United States). The human galectin-3 ELISA is an enzyme-linked immunosorbent assay for the quantitative detection of human galectin-3 (Platinum Elisa, eBioscience, San Diego, CA, United States). The assay was performed measuring the protein in EDTA plasma. Aliquots of serum samples were stored at temperature ranging from 2° to 8°, and the human galectin-3 level were determined after 24 h. Each sample was manually measured, and it has been assayed in duplicate; a calibration curve was built making serial dilution, starting from a value of 25,000 ng/ml to a value of 0.39 ng/ml. The final reading was realized using a specific scanner (DV 990 BV 4/6, N.T. laboratory Rome, Italy). The Presage sST2 assay is a quantitative sandwich monoclonal ELISA in a 96-well microtiter plate format for the measurement of sST2 in serum, EDTA plasma, or heparin plasma. The Presage sST2 assay utilizes two mAbs against ST2. A mouse monoclonal antihuman sST2 antibody is coated onto the surface of the microtiter plate wells and acts as the capture antibody to bind sST2 molecules in the solution. A second mouse monoclonal antihuman sST2 antibody is provided in the solution and functions as the tracer antibody for detecting ST2 molecules that bounded to the capture antibody (Critical Diagnostics, San Diego, CA, United States).

### 2.3. Echocardiography

All patients underwent a cardiologic evaluation and echocardiographic study at baseline, before CRT implantation, and at 1-year follow-up. The responders were defined by the reduction of LV end-systolic volume ≥15% at 1-year follow-up. The echocardiographic evaluation was interpreted and independently reviewed by three senior cardiologists according to the instructions provided by the American Society of Echocardiography (13). The LV volumes and LVEF were calculated using the apical two- and four-chamber views by the Simpson biplane formula. The pulsed-Doppler transmitral flow velocity was used to obtain the early diastolic velocity (E wave), late diastolic velocity (A wave), and their ratio (E/A), and the deceleration time of E wave. The tissue Doppler imaging (TDI) was used to collect the early diastolic myocardial velocity (e’) at the septal and lateral level and the average E/e’ ratio. The M-mode was then used to obtain the values of tricuspid annular plane systolic excursion (TAPSE). The delta (*Δ*) was defined as the difference between the echocardiographic data (LV volumes, LVEF) at baseline and 1-year follow-up.

### 2.4. Outcome definition

The primary clinical outcome was assessed using a composite clinical endpoint consisting of CV mortality and HF hospitalization. CV decease includes death that result from an acute myocardial infarction, sudden cardiac death, HF, stroke, CV procedures, CV hemorrhage, and other CV causes. The secondary outcomes were cardiovascular mortality, HF hospitalizations, and the first episode of sustained rapid ventricular tachyarrhythmias > 180 beats/min detected and terminated or recorded by the CRT device. All such events are routinely registered in our database at each outpatient visit and following consultations in the emergency room of hospital wards. The mean follow-up was 9 ± 2 years.

### 2.5. Statistical analysis

The continuous variables reported as mean ± standard deviation (SD) or as median were compared between patients with CV events and patients without CV events using the Student's *t*-test or non-parametric tests, as appropriate. The *χ*^2^ or Fisher exact test was used to compare non-continuous variables expressed as proportions. The categorical variables reported as percentages were compared between groups using the chi-squared test (or a Fisher exact test when any expected cell count was <5). The predictive parameters of the outcomes were determined by analyzing the receiver operating characteristic (ROC) curves to obtain the best cut-off values. The survival analyses and curves were performed using the Kaplan–Meier method. A Cox regression modeling was performed to assess the factors associated with the composite outcome, CV death or HF hospitalization: multivariate analyses included covariates in a rate of 1:10 with the events recorded. Given the relative low numbers of events at follow-up, we built different prognostic models including at least one clinical, one echocardiographic, and one laboratory parameter. The ones with the highest log-likelihood were then selected. *P*-values are two-sided and considered significant when <0.05. All analyses were performed using IBM SPSS Statistics for Macintosh, Version 26.0 (IBM Corp., Armonk, NY, United States).

## 3. Results

### 3.1. Baseline characteristics, biomarkers, and primary outcome

A total of 86 patients fulfilled the inclusion criteria and were enrolled in the current study. The mean age was 70 ± 9 years, mean QRS duration 165 ± 21 ms, and LVEF 26 ± 6%, and 43% of them had ischemic cardiomyopathy (Table 1). The biomarker levels according to HF etiology (non-ischemic vs. ischemic) are shown in Supplementary Table S1. The patients with non-ischemic etiology showed lower levels of sST2 and better renal function both at baseline and during follow-up compared with patients with ischemic etiology.

**Table 1:** Baseline and follow-up clinical, biomarkers, and echocardiographic characteristics of the population enrolled.

	Total patients, *N* = 86
Baseline	
Age (years)	70 ± 9
Sex female	27 (31)
NYHA functional class	
II	25 (28)
III	56 (66)
IV	5 (6)
Ischemic etiology	37 (43)
Diabetes	26 (30)
Smoke	36 (42)
Dyslipidemia	43 (50)
Hypertension	53 (62)
COPD	13 (15)
AF	21 (24)
QRS duration	165 ± 21
Left ventricular pacing site	
Lateral	59 (68)
Posterolateral	16 (19)
Anterolateral	11 (13)
Biomarkers	
Creatinine (mg/dl)	1.40 ± 0.75
eGFR (ml/min/1.73 m^2^)	55.6 ± 20.0
NT-proBNP (pg/ml)	2,510 ± 4,445
Gal-3 (ng/ml)	27.2 ± 12.4
sST2 (ng/ml)	30.7 ± 11.0
Echocardiographic data	
LVEDV (ml)	210 ± 67
LVESV (ml)	154 ± 54
LVEF (%)	26 ± 6
LVDD (mm)	67 ± 9
LVDS (mm)	54 ± 10
E/A	1.3 ± 0.7
E/e’	16 ± 6
LA area (cm^2^)	24 ± 6
TAPSE (mm)	18.2 ± 4.0
Treatment	
B-blockers	80 (93)
ACEi/ARB	75 (87)
MRA	71 (83)
Loop diuretics	82 (95)
Follow-up	
QRS duration	114 ± 20
Biomarkers	
Creatinine (mg/dl)	1.38 ± 0.55
eGFR (ml/min/1.73 m^2^)	55.5 ± 21.0
NT-proBNP (pg/ml)	2,194 ± 3,856
Gal-3 (ng/ml)	24.2 ± 11.5
sST2 (ng/ml)	26.7 ± 12.2
Echocardiographic data	
LVEDV (ml)	181 ± 77
ΔLVEDV (ml)	−33 ± 34
LVESV (ml)	118 ± 50
ΔLVESV (ml)	−36 ± 41
LVEF (%)	37 ± 11
ΔLVEF (%)	11 ± 11
LVDD (mm)	64 ± 11
LVDS (mm)	51 ± 12
E/e’	14.8 ± 6.4
LA area (cm^2^)	25 ± 6
TAPSE (mm)	18.4 ± 4.0
Treatment	
B-blockers	82 (95)
ACEi/ARB	56 (65)
Sacubitril/valsartan	22 (25)
MRA	73 (85)
Loop diuretics	77 (90)

AF, atrial fibrillation; CV, cardiovascular; HF, heart failure; VA, ventricular arrhythmias; BSA, body surface area; COPD, chronic obstructive pulmonary disease; LVEDV, left ventricular end-diastolic volume; LVESV, left ventricular end-systolic volume; LVEF, left ventricular ejection fraction; LVDD, left ventricular diastolic diameter; LVDS, left ventricular systolic diameter; LA, left atrium; TAPSE, tricuspid annular plane systolic excursion; ACEi, angiotensin-converting-enzyme inhibitors; ARB, angiotensin receptor blockers; MRA, mineralcorticoid receptor antagonist; eGFR, estimated glomerular filtration rate; NT-proBNP, N-terminal portion of the B-type natriuretic peptide; Gal-3, galectin-3; sST2, soluble suppression of tumorigenicity 2.

All values are expressed as absolute number (*n*) and (%) for categorical variables or mean ± standard deviation for continuous variables.

At a median follow-up of 9 ± 2 years, 38 patients (44%) experienced any component of the primary outcome: considering the single outcomes, 33 (38%) were hospitalized for HF and 20 (23%) died. Moreover, 15 (17%) experienced an episode of ventricular arrhythmia. Table 2 shows the differences of the baseline characteristics of the study groups in relation to the composite primary outcome.

**Table 2:** Baseline and follow-up clinical, biomarkers, and echocardiographic characteristics of the patients with CV mortality and HF hospitalization vs. patients without CV events.

	Patients with CV mortality, HF hospitalization *N* = 38	Patients without events *N* = 48	*p*-value
Baseline			
Age (years)	71 ± 8	69 ± 9	0.5
Sex female	10 (26)	17 (35)	0.01
BSA (mq)	1.7 ± 0.14	1.6 ± 0.10	0.09
Ischemic etiology	20 (52)	17 (35)	<0.001
Diabetes	16 (42)	10 (21)	<0.001
Biomarkers			
Creatinine (mg/dl)	1.44 ± 0.48	1.30 ± 0.43	0.007
eGFR (ml/min/1.73 m^2^)	51.5 ± 19	56.7 ± 19	<0.001
NT-proBNP (pg/ml)	2,820 ± 4,778	1,036 ± 928	<0.001
Gal-3 (ng/ml)	34.4 ± 8.2	19.4 ± 8.3	<0.001
sST2 (ng/ml)	37.9 ± 11	23.5 ± 6.9	<0.001
Echocardiographic data			
LVEDV (ml)	238 ± 75	194 ± 58	<0.001
LVESV (ml)	175 ± 60	141 ± 51	<0.001
LVEDV/BSA (ml/mq)	134 ± 31	125 ± 41	<0.001
LVESV/BSA (ml/mq)	127 ± 39	110 ± 33	<0.001
LVEF (%)	26 ± 6	28 ± 5	0.01
LVDD (mm)	71 ± 9	64 ± 8	<0.001
LVDS (mm)	58 ± 9	52 ± 8	<0.001
E wave (cm/s)	86 ± 28	68 ± 23	<0.001
E/A	1.45 ± 0.9	1.04 ± 0.6	<0.001
E/e’	18.8 ± 5	13.4 ± 5	<0.001
LA area (cm^2^)	24 ± 6	23 ± 4	0.01
TAPSE (mm)	16.7 ± 4.1	19.3 ± 3.9	<0.001
Treatment			
B-blockers	35 (92)	45 (93)	0.6
ACEi/ARB	32 (84)	43 (89)	0.9
MRA	31 (82)	40 (83)	0.6
Loop diuretics	36 (94)	46 (95)	0.4
Follow-up			
Biomarkers			
Creatinine (mg/dl)	1.64 ± 0.71	1.17 ± 0.39	<0.001
eGFR (ml/min/1,73 m2)	48.5 ± 24	62.7 ± 21	<0.001
NT-proBNP (pg/ml)	2,858 ± 4,741	963 ± 1,158	<0.001
Gal-3 (ng/ml)	35.3 ± 12.1	18.2 ± 8.1	<0.001
sST2 (ng/ml)	33.4 ± 12	20 ± 9.9	<0.001
Echocardiographic data			
LVEDV (ml)	205 ± 77	158 ± 75	<0.001
ΔLVEDV (ml)	−24 ± 14	−42 ± 53	<0.001
LVESV (ml)	140 ± 55	95 ± 45	<0.001
ΔLVESV (ml)	−25 ± 45	−46 ± 36	<0.001
LVEF (%)	32 ± 10	41 ± 10	<0.001
ΔLVEF (%)	6 ± 11	14 ± 10	<0.001
LVDD (mm)	68 ± 10	62 ± 10	<0.001
LVDS (mm)	56 ± 11	49 ± 11	<0.001
E/e’	17.3 ± 6	12.3 ± 6	<0.001
LA area (cm^2^)	26 ± 6	23 ± 6	<0.001
TAPSE (mm)	16.8 ± 3.7	20.7 ± 3.4	<0.001

CV, cardiovascular; HF, heart failure; VA, ventricular arrhythmias; BSA, body surface area; LVEDV, left ventricular end-diastolic volume; LVESV, left ventricular end-systolic volume; LVEF, left ventricular ejection fraction; LVDD, left ventricular diastolic diameter; LVDS, left ventricular systolic diameter; LA, left atrium; TAPSE, tricuspid annular plane systolic excursion; ACEi, angiotensin-converting-enzyme inhibitors; ARB, angiotensin receptor blockers; MRA, mineralcorticoid receptor antagonist; eGFR, estimated glomerular filtration rate; NT-proBNP, N-terminal portion of the B-type natriuretic peptide; Gal-3, galectin-3; sST2, soluble suppression of tumorigenicity 2.

All values are expressed as absolute number (*n*) and (%) for categorical variables or mean ± standard deviation for continuous variables.

In the group with clinical events, the concentrations of all the biomarkers analyzed were significantly higher, as shown by the mean values of creatinine (1.44 ± 0.48 vs. 1.30 ± 0.43 mg/dl, *p* < 0.001), NT-proBNP (2,820 ± 4,778 vs. 1,036 ± 928 pg/ml, *p* < 0.001), Gal-3 (34.4 ± 8.2 vs. 19.4 ± 8.3 mg/ml *p* < 0.001), and sST2 (37.9 ± 11 vs. 23.5 ± 6.9 ng/ml, *p* < 0.001).

Considering the relative low numbers of events at follow-up, several multivariate analyses including at least one clinical, one echocardiographic, and one laboratory parameter were considered. Table 3 includes some of the multivariate analyses among those showing the highest log-likelihood. Baseline Gal-3 and sST2 cut-off values with the highest AUC at the ROC curve analysis were identified (Gal-3 cut-off: 16.6 ng/ml, AUC: 0.91, *p* < 0.001; sST2 cut-off: 35.6 ng/ml AUC: 0.91, *p* < 0.001) and maintained a strong correlation with the outcome at the multivariate analyses [HR 8.33 (1.88–33.33), *p* = 0.005 and HR 333 (250–1,000), *p* = 0.003, respectively]. In these “prediction models”, E/e’ was also statistically significant; conversely, no clinical variable maintained its correlation with the outcome, including ischemic etiology, as shown in Supplementary Table S2.

**Table 3:** Prediction models with multivariable risk analyses for CV death and HF hospitalization (primary outcome).

Model 1					
Parameter	*p*-value	HR	CI min	CI max	log-likelihood = 88.75
Baseline Gal-3^a^	0.005	8.33	1.88	33.33	
E/e'	0.001	1.22	1.09	1.37	
ΔLVESV	0.095				
Sex	0.068				
Model 2					
Parameter	*p*-value	HR	CI min	CI max	log-likelihood = 51.33
Baseline sST2^b^	0.003	333	250	1,000	
E/e'	0.001	0.72	0.59	0.88	
*Δ*LVESV	0.130				
Sex	0.276				
Model 3					
Parameter	*p*-value	HR	CI min	CI max	log-likelihood = 93.02
eGFR FU	0.040	0.84	0.74	0.91	
E/e'	0.001	1.20	1.08	1.33	
ΔLVESV	0.497				
Sex	0.088				
Model 4					
Parameter	*p*-value	HR	CI min	CI max	log-likelihood = 78.88
ΔGal-3	0.001	1.25	1.09	1.43	
E/e’	0.006	1.18	1.05	1.33	
ΔLVESV	0.595				
Sex	0.226				

eGFR, estimated glomerular filtration rate; LVESV, left ventricular end-systolic volume; LVEF, left ventricular ejection fraction; Gal-3, galectin-3; sST2, soluble suppression of tumorigenicity 2; FU, follow-up.

^a^ Gal-3 ≥ 16.6 pg/ml.

^b^ sST2 ≥ 35.6 ng/ml.

**Figure 1:**
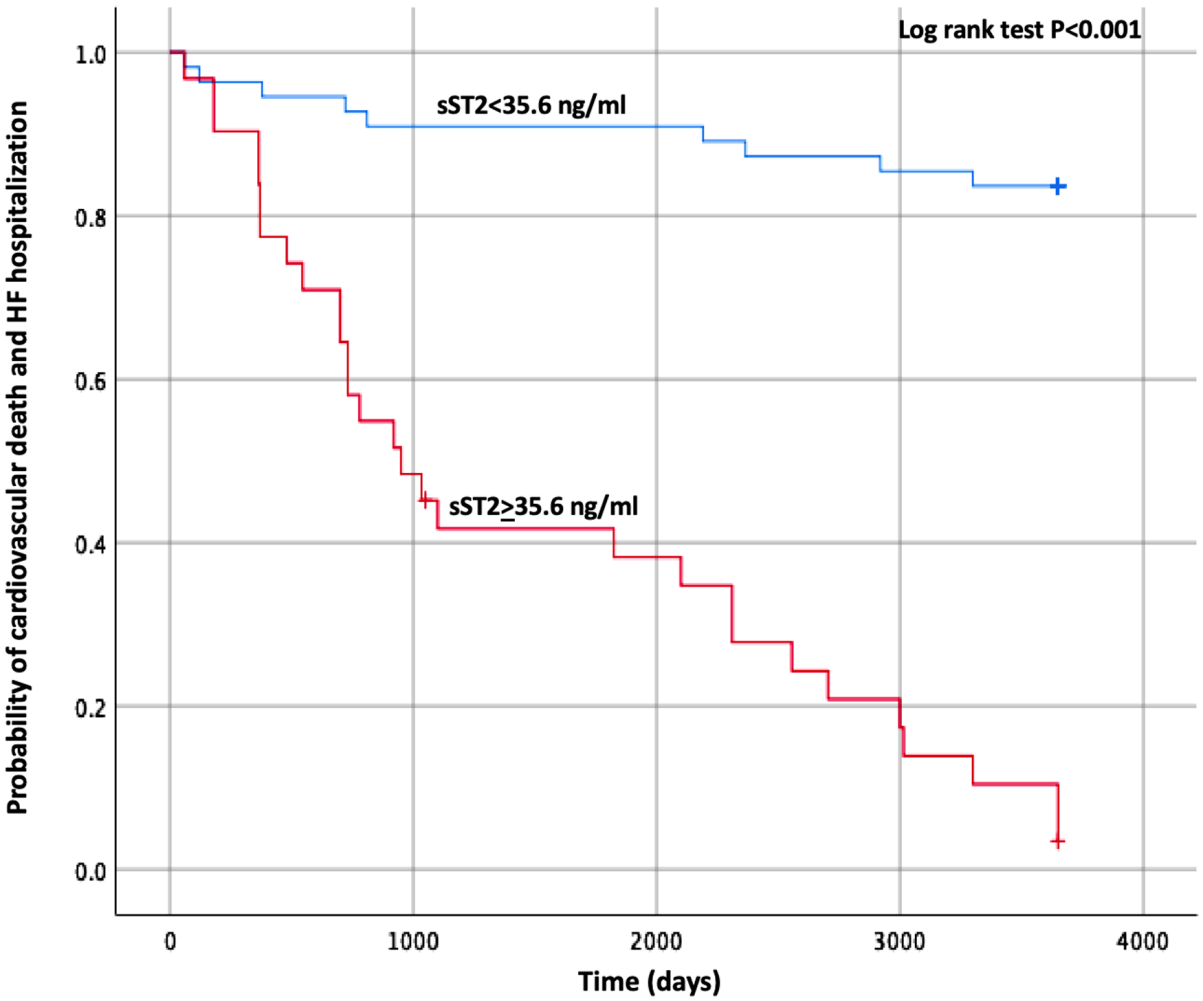
Kaplan–Meier estimates of the cumulative probability of CV death and HF hospitalization by ST2 cut-off. CV, cardiovascular; HF, heart failure; ST2, suppression of tumorigenicity 2.

**Figure 2:**
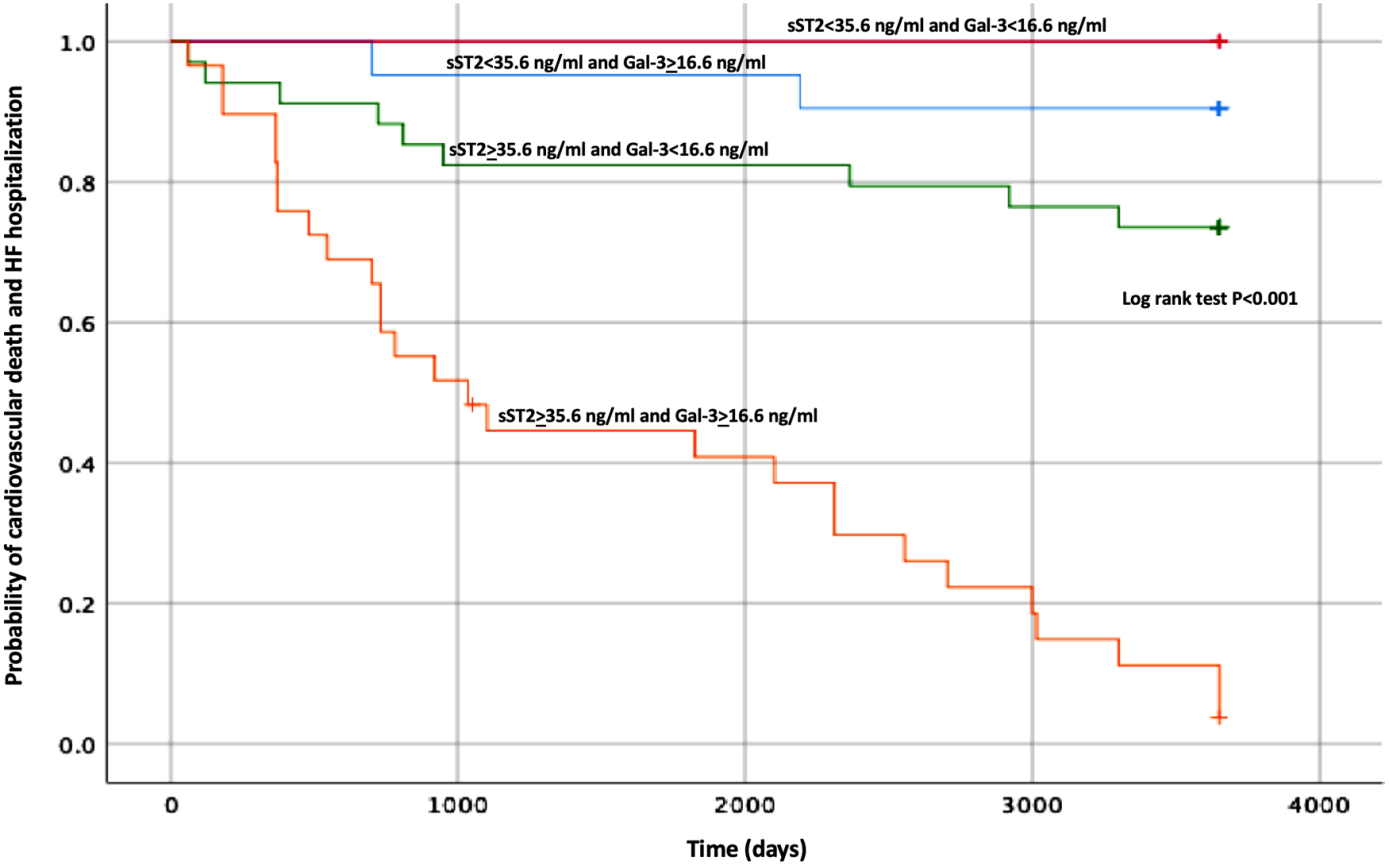
Kaplan–Meier estimates of the cumulative probability of CV death and HF hospitalization by Gal-3 and ST2 levels. CV, cardiovascular; HF, heart failure; ST2, suppression of tumorigenicity 2; Gal-3, galectin-3.

The Kaplan–Meier curve built with the same cut-off values of sST2 is shown in Figure 1. The survival curves were then created using the combination of the cut-off values of sST2 and Gal-3 found in our analysis. As displayed in Figure 2, the patients with both high baseline sST2 and Gal-3 had the lowest survival probability.

NT-proBNP was not significantly related with the composite outcome when considered singularly in various prediction models, but the parameter obtained by its combination with sST2 (both considered as categorical variables) proved to be significant [HR 7.69 (3.13–20), *p* < 0.001, log-likelihood = 56.16] and showed a high prediction performance (AUC 0.85).

The laboratory values obtained at 12 months confirmed the same trend presented at baseline, with higher levels of all cardiac biomarkers in the patients with CV events. At the multivariate analyses, sST2 [HR 1.15 (1.08–1.22), *p* < 0.001] and the ΔGal-3 [HR 1.26 (1.10–1.43), *p* ≤ 0.001] maintained their prognostic value at follow-up (Table 3). Interestingly, the baseline eGFR values did not significantly correlate with the composite outcome, as opposed to the values obtained at 1-year follow-up [HR: 0.84 (0.74–0.91), *p* = 0.04].

### 3.2. Predictive role of biomarkers and secondary outcomes

**Figure 3:**
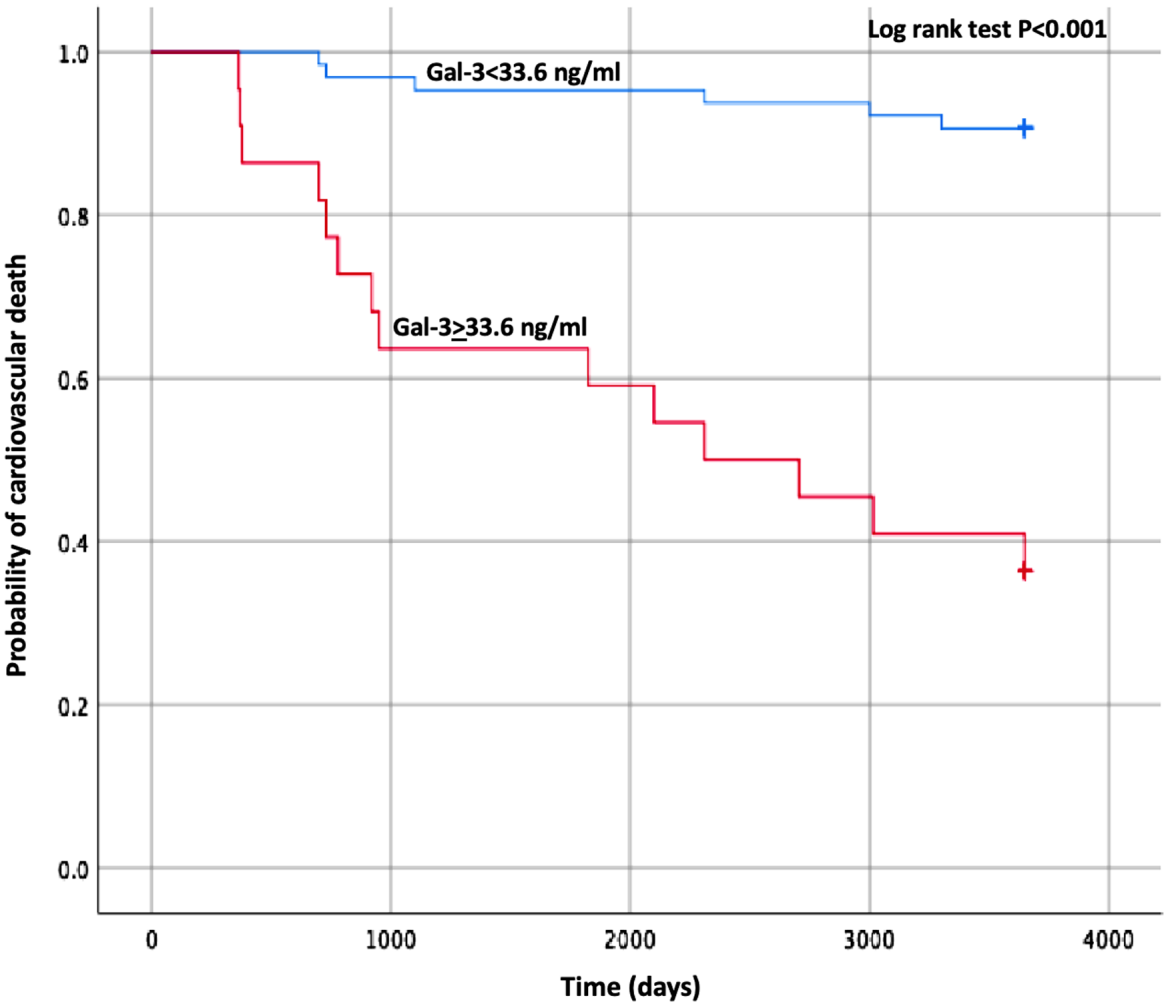
Kaplan–Meier estimates of the cumulative probability of cardiovascular death by Gal-3 cut-off. Gal-3, galectin-3.

Concerning the secondary endpoints, baseline Gal-3 and sST2 maintained their significant association with both HF hospitalizations and CV mortality alone at the multivariate analyses, as shown in Supplementary Table S3, S4. The best predictor of CV mortality was Gal-3 with a cut-off value of 33.6 pg/ml (AUC 0.91 *p* < 0.001), and the relative Kaplan–Meier curve is shown in Figure 3.

As for the composite outcome, even if NT-proBNP alone was not significant, the parameter obtained by its combination with baseline sST2 (AUC 0.88, *p* < 0.001) showed good outcome prediction at the multivariate analysis [HR for HF hospitalizations 3.23 (1.89–4.35), *p* < 0.001, log-likelihood = 58.26; HR for CV mortality 3.33 (1.39–5.88), *p* = 0.010, log-likelihood = 59.37].

When biomarkers were evaluated at 1 year, the prediction model built with ΔGal-3 was predictive for the single components of secondary outcomes [HR for HF hospitalizations 1.19 (1.07–1.33), *p* = 0.001; HR for CV mortality 1.14 (1.04–1.26), *p* = 0.005]. The eGFR at follow-up maintained its prognostic role for cardiovascular mortality [HR 0.84 (0.80–0–90), *p* = 0.002].

Finally, no single predictor for the outcome of ventricular arrhythmias was significant at the multivariate analysis.

### 3.3. LV dimensions, CRT response, and outcomes

The difference between the left ventricular end-systolic volume (LVESV) at baseline and follow-up (*Δ*LVESV) did not correlate with primary and secondary outcome in all prognostic models when including biomarkers at the multivariate analyses, as shown in Table 3 and Supplementary Tables S2–S4. Accordingly, the echocardiographic definition of CRT response did not relate with the single components of the composite outcome in various prediction models and, among the total of 52 (62%) patients who showed LV reverse remodeling and were considered responders to CRT, no difference in terms of events rate was recorded.

## 4. Discussion

### 4.1. Biomarkers and outcome

In this paper concerning HFrEF patients undergoing CRT implantation, a significant correlation between biomarkers of myocardial inflammation, congestion, and fibrosis, together with renal function and the composite long-term outcome of HF hospitalization and CV mortality was found. Gal-3 and sST2 showed the highest power in the prediction models for CV death and HF hospitalization, also when analyzed as single endpoints. This finding supports the theory that inflammation and fibrosis can contribute to the course of the disease even when HF reaches advanced stages, indicating an ongoing myocardial damage and portending poor prognosis (14).

Although NT-proBNP, sST2, Gal-3, and eGFR, when considered individually, have already been demonstrated to have a prognostic role in HF (15–17), to the best of our knowledge, a combination of these parameters and its variations during the course of the disease were not tested in the candidates to CRT implantation in the long-term follow-up.

The sST2 was included in the Biomarker CRT score, developed in a sub-analysis of the SMART-AV trial, due to its additive predictive value of CRT response when considered against a composite of clinical variables (18). Its concentrations have been shown to predict sudden cardiac death in patients with HFrEF and provided complementary information to NT-proBNP (19). Moreover, serial measurements of sST2 provided incremental information to baseline levels, reflecting changes in myocardial remodeling over time and an increased risk of CV death (20).

Similarly, Gal-3 is a soluble beta-galactoside-binding lectin that has been related to inflammation and fibroblast activation; its effect on myocardial fibrosis, CV stiffness, and immune response modulation seems to determine pathological myocardial remodeling (21). High Gal-3 values have been related with CRT response at 6 months and with CV outcome at 48 months. Serial measurements have shown a prognostic role in acute HF, independently from BNP values (22). In a sub-analysis of CARE-HF, Gal-3 was an independent predictor of death from any cause or an unplanned hospitalization for a major CV event, even if it did not predict the response to CRT if considered as a separated outcome (23). Interestingly, in our population, the patients who met the primary outcome also showed higher Gal-3 levels at 1-year follow-up, and the ΔGal-3 was a prognostic marker at the prediction model, in line with the previous findings by Van Vark et al. (24).

When it comes to HF, heart and kidney functions are strictly intertwined. A renal dysfunction is very common in HFrEF, and it is acknowledged as a powerful predictor of survival (25). From a pathophysiological point of view, several mechanisms explain renal involvement in cardiac diseases, mainly attributable to renal congestion due to elevated venous pressure, decreased cardiac output, and activation of neurohormonal system (26). It has been described that the slight improvement in cardiac output after CRT may be associated with a concurrent improvement in renal function (27, 28). In our analysis, we confirm that the patients with adverse CV outcome show a significant decline of eGFR at follow-up compared with the patients without CV events. Moreover, eGFR is able to predict the absolute risk for adverse cardiac events. Maaten et al. demonstrated that the patients with chronic kidney disease undergoing CRT implantation, while experiencing a reverse remodeling in a lesser extent than those patients without renal dysfunction, also derive benefit on outcome at a lesser degree of remodeling. This could be related to the underlying pathogenesis of the renal dysfunction, such as nephrosclerosis, which is unlikely to respond to hemodynamic improvement (29).

### 4.2. Response to CRT and outcome

At follow-up, *Δ*LVESV and the reduction of more than 15% of LVESV did not show a significant relation with the outcome in our population. This finding seems to contrast with the large actual attention for the so-called CRT “response,” but lines up with a recent ESC position statement, which questions this arbitrary definition (30). Historically, the interest in literature for the research of variables to identify the patients who are less likely to benefit from CRT has always been alive. Also, a uniform way to define the desirable echocardiographic “response” to CRT is lacking, and echo improvement has been shown to be variable among different etiologies of HF. In fact, it has been argued that a binary definition of response underestimates the true benefits of CRT and that similar attention has not been posed to select patients for drug therapy. We challenge the idea that the selection of CRT candidates should be limited in base of the underlying etiology: while it is true that the patients with an ischemic etiology manifest less reverse remodeling, it should also be noticed that they have an equal relative risk reduction after CRT for HF admission and death as the non-ischemic group. Moreover, such a simple and cautious approach has resulted in a well-known undertreatment of dyssynchrony, preventing the patients to take advantage of a device that demonstrated to reduce morbidity and mortality (31). A lack of improvement in LVEF or in the symptoms of the patients (also considering the limitations of this evaluation) is not considered a good reason to withdraw one of the “drugs pillars” and accordingly should not be interpreted as a failure of CRT. The term “disease modification” (that may even imply a mere stabilization) should therefore replace the term “response” (32). Accordingly, our results corroborate the role of the laboratory values in CRT recipients, beyond the technical parameters used to define the efficacy of resynchronization. Importantly, the levels of these biomarkers, namely, sST2 and Gal-3, together with renal function, maintained their prognostic power also at 1-year follow-up. This should encourage clinicians to serially assess those values, especially when considering that many other parameters do not hold the same significance in advanced HF stages.

In our cohort, the risk stratification models incorporating one biomarker and E/e’ identified the patients at risk for CV outcome, confirming that the patients with higher left ventricular filling pressure (LVFP) at baseline before the device implantation show a worse prognosis. E/e’ is the most robust echocardiographic surrogate of an elevated LVFP, and several validation studies have confirmed the prediction of normal and abnormal LVFP when E/e’ ratio was <8 or >15 (33, 34). Elevated values of E/e’ ratio related to HF progression and worse prognosis as a consequence of an increased myocardial stiffness (35). Our data reproduce the findings of the REVERSE trial, where E/e’ ratio was associated with the endpoints of mortality and a new or recurrent HF in CRT recipients (36).

In summary, our results suggest that the laboratory parameters related to fibrosis production and extracellular matrix deposition, together with the concordant increase in echocardiographic surrogates of wall rigidity and chamber stiffness, are linked to an unfavorable outcome in CRT patients.

Our study comes with several limitations. All data were collected retrospectively from our single center, allowing the achievement of a small sample size with low cardiovascular events. However, we included an accurately screened population undergoing CRT implantation, following the indication of the latest guidelines. Gal-3 often increases in renal failure and chronic inflammatory diseases. Moreover, the so-called “response” to CRT depends on many different parameters: a role of underlying etiology, percentage of biventricular pacing, loss of LV capture, and compliance to medical therapy cannot be ruled out. In addition, notwithstanding that efforts should be made to optimize the efficacy of CRT capture after implantation, this does not affect the main finding of our study concerning the correlation between the biomarkers and the outcomes in these patients.

A total of four patients experienced the primary endpoint in the first year after the implantation, hence the correlation between echocardiographic and laboratory parameters at 1-year follow-up, and the outcome do not apply for them.

In conclusion, our study showed how, in a population of HFrEF patients implanted with CRT, a combined evaluation of biomarkers of cardiac inflammation, fibrosis, and renal function correlated with the combined outcome of CV death and HF hospitalization, as opposite to the echocardiographic definition of CRT response. The current findings cannot be extended to all HF patients with different etiologies and need to be confirmed in larger multi-center studies. However, despite the potential confounders, our results encourage clinicians to serially assess the levels of cardiac biomarkers to add significant prognostic implication.

## Data Availability

The original contributions presented in the study are included in the article/Supplementary Material, further inquiries can be directed to the corresponding author.
