# Supplementary material for: The role of fibrosis, inflammation, and congestion biomarkers for outcome prediction in candidates to cardiac resynchronization therapy: is “response” the right answer?
Source: Front Cardiovasc Med. 2023 Jun 12;10:1180960. doi: 10.3389/fcvm.2023.1180960 (PMC10291081; doi:10.3389/fcvm.2023.1180960)
Supplement: Supplementary file 1 [file Table1.docx]

|  | **Non-ischemic aetiology (N=49)** | **Ischemic aetiology (N=37)** |  |
| --- | --- | --- | --- |
| ***Baseline*** |  |  |  |
|  |  |  |  |
| **Creatinine (mg/dl)** | **1.23±0.32** | **1.52±0.52** | **0.007** |
| **eGFR (ml/min/1,73 m2)** | **56.5±17** | **48.9±16** | **<0.001** |
| **NT-proBNP (pg/ml)** | **2015±2811** | **3156±5213** | **<0.001** |
| **Gal-3 (ng/ml)** | **26.1±11.9** | **26.3±10.6** | **0.6** |
| **sST2 (ng/ml)** | **28.9±8.9** | **32.3±13** | **0.03** |
| ***Follow-up*** |  |  |  |
|  |  |  |  |
| **Creatinine (mg/dl)** | **1.19±0.44** | **1.60±0.62** | **0.001** |
| **eGFR (ml/min/1,73 m2)** | **61.7±23** | **47.6±17** | **<0.001** |
| **NT-proBNP (pg/ml)** | **1451±1606** | **2387±4424** | **<0.001** |
| **Gal-3 (ng/ml)** | **21.8±9.4** | **25.8±12.2** | **0.01** |
| **sST2 (ng/ml)** | **24.5±11** | **29±13** | **0.02** |
