# Supplementary material for: The role of fibrosis, inflammation, and congestion biomarkers for outcome prediction in candidates to cardiac resynchronization therapy: is “response” the right answer?
Source: Front Cardiovasc Med. 2023 Jun 12;10:1180960. doi: 10.3389/fcvm.2023.1180960 (PMC10291081; doi:10.3389/fcvm.2023.1180960)
Supplement: Supplementary file 2 [file Table2.docx]

| **Supplementary table 2 – Prediction models with multivariable risk analyses including ischemic aetiology for CV death and HF hospitalization** | | | | | |
| --- | --- | --- | --- | --- | --- |
| **Model 1** |  |  |  |  |  |
| **Parameter** | **p-value** | **HR** | **CI min** | **CI max** | **log likelihood=**46.33 |
| Baseline sST2* | <0.001 | 333 | 23 | 1000 |  |
| E/e' | 0.001 | 1.39 | 1.15 | 1.69 |  |
| ΔLVESV | 0.130 |  |  |  |  |
| Ischemic aetiology | 0.811 |  |  |  |  |
|  |  |  |  |  |  |
| **Model 2** |  |  |  |  |  |
| **Parameter** | **p-value** | **HR** | **CI min** | **CI max** | **log likelihood=**88.75 |
| Baseline Gal-3** | 0.005 | 8.33 | 1.92 | 50 |  |
| E/e' | 0.001 | 1.23 | 1.10 | 1.37 |  |
| ΔLVESV | 0.095 |  |  |  |  |
| Ischemic aetiology | 0.694 |  |  |  |  |
|  |  |  |  |  |  |
| **Model 3** |  |  |  |  |  |
| **Parameter** | **p-value** | **HR** | **CI min** | **CI max** | **log likelihood=**77.88 |
| ΔGal-3 | 0.001 | 1.25 | 1.09 | 1.43 |  |
| E/e' | 0.006 | 0.85 | 0.75 | 0.95 |  |
| ΔLVESV | 0.060 |  |  |  |  |
| Ischemic aetiology | 0.923 |  |  |  |  |
|  |  |  |  |  |  |
|  |  |  |  |  |  |
| **Model 4** |  |  |  |  |  |
| **Parameter** | **p-value** | **HR** | **CI min** | **CI max** | **log likelihood=**93.02 |
| eGFR FU | 0.040 | 0.80 | 0.71 | 0.91 |  |
| E/e' | 0.001 | 0.83 | 0.75 | 0.93 |  |
| ΔLVESV | 0.427 |  |  |  |  |
| Ischemic aetiology | 0.588 |  |  |  |  |

* sST2 ≥35.6 ng/ml

** Gal-3 ≥16.6 pg/ml

Estimated Glomerular Filtration Rate (eGFR), Left ventricular end systolic volume (LVESV), left ventricular ejection fraction (LVEF).

follow-up (FU)
