# Supplementary material for: The role of fibrosis, inflammation, and congestion biomarkers for outcome prediction in candidates to cardiac resynchronization therapy: is “response” the right answer?
Source: Front Cardiovasc Med. 2023 Jun 12;10:1180960. doi: 10.3389/fcvm.2023.1180960 (PMC10291081; doi:10.3389/fcvm.2023.1180960)
Supplement: Supplementary file 3 [file Table3.docx]

| **Supplementary table 3 – Prediction models for HF hospitalization** | | | | | |
| --- | --- | --- | --- | --- | --- |
| **Model 1** |  |  |  |  |  |
| **Parameter** | **p-value** | **HR** | **CI min** | **CI max** | **log likelihood=**61.54 |
| Baseline sST2* | <0.001 | 28.77 | 7.57 | 109.35 |  |
| E/e' | 0.011 | 1.20 | 1.04 | 1.39 |  |
| ΔLVESV | 0.558 |  |  |  |  |
| Age | 0.820 |  |  |  |  |
|  |  |  |  |  |  |
| **Model 2** |  |  |  |  |  |
| **Parameter** | **p-value** | **HR** | **CI min** | **CI max** | **log likelihood=**76.09 |
| Baseline Gal-3** | <0.001 | 10.72 | 3.47 | 33.12 |  |
| E/e' | 0.011 | 1.16 | 1.04 | 1.31 |  |
| ΔLVESV | 0.582 |  |  |  |  |
| Age | 0.485 |  |  |  |  |
|  |  |  |  |  |  |
| **Model 3** |  |  |  |  |  |
| **Parameter** | **p-value** | **HR** | **CI min** | **CI max** | **log likelihood=**88.90 |
| sST2 FU | <0.001 | 10.98 | 3.76 | 32.01 |  |
| FE | 0.230 |  |  |  |  |
| ΔLVESV | 0.862 |  |  |  |  |
| Age | 0.590 |  |  |  |  |
|  |  |  |  |  |  |
| **Model 4** |  |  |  |  |  |
| **Parameter** | **p-value** | **HR** | **CI min** | **CI max** | **log likelihood=**80,60 |
| ΔGal-3 | 0.001 | 1.19 | 1.07 | 1.33 |  |
| E/e' | 0.001 | 1.22 | 1.09 | 1.36 |  |
| ΔLVESV | 0.257 |  |  |  |  |
| Age | 0.179 |  |  |  |  |

* sST2≥33.7 pg/ml

** Gal-3≥29.2 pg/ml

Left ventricular end systolic volume (LVESV), left ventricular ejection fraction (LVEF), follow-up (FU)
