# Supplementary material for: The role of fibrosis, inflammation, and congestion biomarkers for outcome prediction in candidates to cardiac resynchronization therapy: is “response” the right answer?
Source: Front Cardiovasc Med. 2023 Jun 12;10:1180960. doi: 10.3389/fcvm.2023.1180960 (PMC10291081; doi:10.3389/fcvm.2023.1180960)
Supplement: Supplementary file 4 [file Table4.docx]

| **Supplementary table 4 – Prediction models for CV mortality** | | | | | |
| --- | --- | --- | --- | --- | --- |
| **Model 1** |  |  |  |  |  |
| **Parameter** | **p-value** | **HR** | **CI min** | **CI max** | **log likelihood=**58.58 |
| Baseline sST2* | <0.001 | 31.25 | 7.58 | 125 |  |
| E/e' | 0.260 |  |  |  |  |
| ΔLVESV | 0.853 |  |  |  |  |
| Age | 0.689 |  |  |  |  |
|  |  |  |  |  |  |
| **Model 2** |  |  |  |  |  |
| **Parameter** | **p-value** | **HR** | **CI min** | **CI max** | **log likelihood=**62.84 |
| Baseline Gal-3** | <0.001 | 18.87 | 5.29 | 66.67 |  |
| E/e' | 0.527 |  |  |  |  |
| ΔLVESV | 0.895 |  |  |  |  |
| Age | 0.884 |  |  |  |  |
|  |  |  |  |  |  |
| **Model 3** |  |  |  |  |  |
| **Parameter** | **p-value** | **HR** | **CI min** | **CI max** | **log likelihood=**76.90 |
| ΔGal-3 | 0.005 | 1.14 | 1.04 | 1.26 |  |
| E/e' | 0.138 |  |  |  |  |
| ΔLVESV | 0.393 |  |  |  |  |
| Age | 0.608 |  |  |  |  |
|  |  |  |  |  |  |
|  |  |  |  |  |  |
| **Model 4** |  |  |  |  |  |
| **Parameter** | **p-value** | **HR** | **CI min** | **CI max** | **log likelihood=**64.30 |
| eGFR FU | 0.002 | 0.84 | 0.80 | 0.90 |  |
| FE | 0.036 | 0.87 | 0.77 | 0.99 |  |
| ΔLVESV | 0.921 |  |  |  |  |
| Age | 0.749 |  |  |  |  |

* sST2≥37.5 pg/ml

** Gal-3≥33.6 pg/ml

Estimated Glomerular Filtration Rate (eGFR), Left ventricular end systolic volume (LVESV), left ventricular ejection fraction (LVEF), follow-up (FU)
